# First Case Report: Treatment of the Facial Warts by Using *Myrtus communis* L. Topically on the Other Part of the Body

**DOI:** 10.5812/ircmj.13565

**Published:** 2014-02-05

**Authors:** Mohamad Bagher Minaei, Elham Ghadami Yazdi, Mohamad Ebrahim Zadeh Ardakani, Fataneh Hashem Dabaghian, Ali Mohammad Ranjbar, Mohamad Rastegari, Ali Ghadami Yazdi

**Affiliations:** 1 Research Deputy of Traditional Medicine, Tehran University of Medical Sciences, Tehran, IR Iran; 2 Faculty of Dermatology, Shahid Sadughi University of Medical Sciences, Yazd, IR Iran; 3 Research Institute for Islamic and Complementary Medicine, Iran University of Medical Sciences, Tehran, IR Iran; 4 Faculty of Traditional Medicine, Department of Traditional Pharmacy, Tehran University of Medical Sciences, Tehran, IR Iran; 5 Faculty of Pharmacy, Department of Pharmacognosy, Shahid Sadughi University of Medical Sciences, Yazd, IR Iran; 6 Payame Noor University, Yazd Branch, Yazd, IR Iran; 7 Faculty of Anesthesiology, Shahid Sadughi University of Medical Sciences, Yazd, IR Iran

**Keywords:** Medicine, Traditional, Warts, *Myrtus*, face

## Abstract

**Introduction:**

Wart is a contagious dermal disease with different types. Wart has long-term treatment with symptoms of multiple relapses, which involve larger surfaces. It has no definite medical treatment in traditional medicine and the provided treatments encounter restrictions and side effects especially in the facial warts.

**Case Presentation:**

Iranian traditional medicine (ITM) has provided different, economic, and low cost treatments for warts. One therapeutic method is using *Myrtus communis* L. (Myrtle) topically. The goal of this study is to investigate the efficacy of Myrtle as a method of ITM. In this study, we present two patients with common warts. They are from Iran and live in Yazd. They were taken Myrtle topically on their body but not on their faces.

**Conclusions:**

The facial warts of both cases have completely cured by using Myrtle. We hypothesized that Myrtle not only have antiviral effects but also may have a systemic impression. It can use topically on a part of body with influence on the other parts. Myrtle is especially useful for facial warts. These two cases highlighted a new method for treatment of common warts especially facial warts and it needs more investigations.

## 1. Introduction

Wart is a contagious dermal disease and has different types. Common warts are usually exophytic, multiple, irregular, rough nodules that show a variety of clinical patterns at different sites of trauma, particularly on fingers, also on abraded skin surfaces (1). Wart has long-term treatment with symptoms of multiple relapses which involve larger surfaces. It has no definite medical treatment in traditional medicine and the provided treatments have restrictions and side effects especially in the facial warts (1-4). Dermal diseases are important in ITM. The wart in ITM literature is known as suloul. There are different types and causes of Suloul. The causes of suloul are sauda, balgam or both. ITM is based on the principle of humeral balance. The principle states that each individual has a different humeral constitution, built from the 4 basic humors (akhlat): Dam (Blood), Balgam (Phlegm), Safra (Yellow Bile) and Sauda (Black Bile). Health is defined as a well-balanced proportion of the humeral fluids in the body while sickness is a condition where the humeral composition is imbalanced or when the qualitative change occurs. The natural balance of the humeral constitution in the body is regulated by a power of self-preservation or adjustment called "Quwwate-Muddabera" or "tabiyat" which means the nature. The practice of ITM helps bring Quwwate-Muddabera or tabiyat to an optimum level at the onset of disease to restore humeral balance and regain a healthy condition. So there are several treatments in ITM, depending on the cause of warts. The treatments may be generally, topically or both of them. Some treatments are inexpensive and low risk. One method of therapy is the topically use of Myrtle (5).

Myrtle is recognized as Aas and its berries are known as "Habb-ul-Aas". It is often grown for its attractive foliage, flowers and berries. Its berries, leaves as well as essential oil are frequently used for various (6). This plant has therapeutic effects on more diseases. It has bitterness along with astringency and some sweetness. It has some coldness because of its astringency. It has an earthy and rarefied substance. It has some mild hotness but the coldness is dominant. Its astringency excels its coldness. Its coldness is in the first degree and the dryness in the second degree. Its astringency is more dominant than its coldness. It is a dissolving, astringency drug and it strengthens the spirit. It has not any important side effects in ITM, in conjunction with the proper administration of designated therapeutic dosages (7-9). In Classic Medicine, Myrtle Shown anti-inflammatory effects (10), the anti-microbial activity of Myrtle on *Escherichia coli*, *Staphylococcus aureus*, *Pseudomonas aeruginosa*, *P. vulgaris*, *P. mirabilis*, *Klebsiella aerogenes*, *Salmonella typhi* and *S. shigiella* has been determined (11). Anti-fungal activity of the essential oil was also studied against Aspergillus which was found effective against all isolates (11).

Myrtle has more effects. From the time immemorial, plants have been extensively used as curative agents for a variety of ailments. Extensive survey revealed that Myrtle has a long history of traditional use for wide range of diseases. Many of the traditional uses have been validated by scientific researches. A number of phytochemicals isolated from various plants like flavonoids, coumarins, tannins, etc. have shown a variety of pharmacological activities like anti-diarrheal, anti-ulcer, anti-diabetic, anti-hypertensive, anti-oxidant, anti-microbial, anti-mutagenic, etc. in various clinical and pharmacological trials (6). We study on wart and its treatments in ITM. We selected Myrtle because of the above mentioned reasons. Moreover, the plant of Myrtle is accessible for us.

## 2. Case Presentation

We present two patients with common warts. Both of them were Iranian girls living in Yazd city. They used Myrtle leaves solution as a remedy on their warts but not on their facial warts. It comprised of one part leaves of Myrtle and two parts of water. They used it twice a day for 20 days. This study was started on March 2013. One of these cases is a 10-year-old girl who presented with a 3-year history of common warts that gradually appearing on her two hands. We measured the number of warts by observation and the large diameter of each wart by ruler in millimeter. She had 16 warts on her 5 fingers of both hands and 6 warts on her face area between her nose and mouth. The large diameter of warts on her hands was less than 10 millimeters and on her face was less than 5 millimeters. Her warts were treated completely within a 20-day course of Myrtle therapy just topically on her hands and then she appeared to have had a complete response, not only on her hands but also on her face. The warts on her face cured completely without using Myrtle on those regions.

The other one is a 12-year-old girl who presented with a 2-year history of a common wart on her neck and a 2-month history of a common wart on lateral angle of her left eye. The diameters of warts were 1 × 1 × 1 millimeter. She was taken a 20-day course of Myrtle therapy just topically on her neck. She appeared to have had a complete response on her face but her neck appeared to have had a near- complete positive result. Although warts may relapse after treatment but none of them had relapse after 6 months. We used ruler to measure the largest diameter of each wart. Although measuring the size of the warts by ruler might have bias but the facial warts were completely disappeared. By the way, we took photographs with digital camera before and after the treatment.

## 3. Conclusions

Wart is contagious and self-inoculated lesion and may relapse after treatment. It has no definite traditional medical treatment and the provided treatments encounter restrictions and side effects especially in cases with facial warts (1-3). We hypothesize that Myrtle may have an anti-viral effect and it may also have systemic effect according to its characteristics. It can strengthen Quwwate-Muddabera that is a concept in ITM, which seems that immune system is a part of it. Myrtle may use topically on a part of a body and show an effect on the other parts. These two cases highlight a new method for treatment of common warts especially facial warts and it needs more investigationsb (Table 1). 

**Table 1.** Variable of Our Cases

Patient	Age, y	Gender	Location of Warts	The Number of Warts. Day 0	The Large Diameter of the Warts. Day 0	The Number of the Warts. Day 20	The Large Diameter of the Warts. Day 20
**1**	10	female	Face, Fingers of two hands	22	< 10 mm	0	0
**2**	12	female	Face/ neck	2	1 mm	1	< 1 mm

In recent years, the emphasis of researches has been on the utilization of traditional medicine that has a long and proven history of treating various ailments. In this regard, further studies are required to be carried out on Myrtle due to its potential in preventing and treating different diseases. The positive points of our study are as follow: 1. We got in touch with our patients every three weeks. 2. We took digital photos before and after treatment. Our study limitations are that the form of the drug that was difficult to use. It is better to prepare more suitable shape of the drug. For example tape of Myrtle may be better to use.
